# Marsupial tammar wallaby delivers milk bioactives to altricial pouch young to support lung development

**DOI:** 10.1016/j.mod.2016.08.004

**Published:** 2016-11

**Authors:** Vengamanaidu Modepalli, Lyn A. Hinds, Julie A. Sharp, Christophe Lefevre, Kevin R. Nicholas

**Affiliations:** a Department of Ecology, Evolution and Behavior, Institute of Life Science, Hebrew University, Edmund J Safra Campus, Jerusalem, Israel; b CSIRO Ecosystem Sciences, GPO Box 1700, Canberra, ACT 2601, Australia; c School of Medicine, Deakin University, Pigdons Road, Geelong, VIC, Australia; d Walter Eliza Hall Institute, 1G Royal Parade, Parkville, Victoria 3052, Australia

**Keywords:** Lung development, Evolution, Marsupials, Lactation, Milk

## Abstract

Our research is exploiting the marsupial as a model to understand the signals required for lung development. Marsupials have a unique reproductive strategy, the mother gives birth to altricial neonate with an immature lung and the changes in milk composition during lactation in marsupials appears to provide bioactives that can regulate diverse aspects of lung development, including branching morphogenesis, cell proliferation and cell differentiation. These effects are seen with milk collected between 25 and 100 days postpartum. To better understand the temporal effects of milk composition on postnatal lung development we used a cross-fostering technique to restrict the tammar pouch young to milk composition not extending beyond day 25 for 45 days of its early postnatal life. These particular time points were selected as our previous study showed that milk protein collected prior to ~ day 25 had no developmental effect on mouse embryonic lungs in culture. The comparative analysis of the foster group and control young at day 45 postpartum demonstrated that foster pouch young had significantly reduced lung size. The lungs in fostered young were comprised of large intermediate tissue, had a reduced size of airway lumen and a higher percentage of parenchymal tissue. In addition, expression of marker genes for lung development (BMP4, WNT11, AQP-4, HOPX and SPB) were significantly reduced in lungs from fostered young. Further, to identify the potential bioactive expressed by mammary gland that may have developmental effect on pouch young lungs, we performed proteomics analysis on tammar milk through mass-spectrometry and listed the potential bioactives (PDGF, IGFBP5, IGFBPL1 and EGFL6) secreted in milk that may be involved in regulating pouch young lung development. The data suggest that postnatal lung development in the tammar young is most likely regulated by maternal signalling factors supplied through milk.

## Highlights

• The temporal effects of milk composition on lung development is well demonstrated.
• Fostered PY lungs were delayed in development due to change in milk composition.
• Tammar milk secretes bioactives involved in lung development.

## 1 Introduction

In mammals the lung has evolved as a respiratory organ to exchange gases immediately after birth (Philip, 1977). In eutherians the majority of lung development occurs during intrauterine life. The lungs of new born are at an alveolar stage of development and the key changes that occur during early postnatal life include increased alveolar number and maturation of microvasculature (Burri, 2006). Respiratory complications are frequently seen in premature infants, largely due to the incomplete development of lung (Burri, 1984). In contrast to eutherians, the marsupial young is born at an altricial stage after a short gestation and the major development occurs postnatally (Nicholas et al., 1997, Renfree, 2006, Tyndale-Biscoe and Renfree, 1988). New born marsupials are similar in development to a late eutherian foetus and the immature lung is required to develop rapidly to become fully functional. Studies in marsupial species including the Julia Creek dunnart (*Sminthopsis douglasi*) (Frappell and Mortola, 2000), tammar wallaby (*Macropus eugenii*) and gray short tailed opossum (*Monodelphis domestica*) (Szdzuy et al., 2008) have shown the lungs of newborns are comprised of a small number of large air sacs providing insignificant surface area for respiration, and the lungs are considered as functionally immature (Mess and Ferner, 2010). Therefore these marsupial newborn have adopted a unique respiratory mechanism to perform partial gaseous exchange through skin during early postnatal development to meet their oxygen demand (Frappell and Mortola, 2000, Mortola et al., 1999).

In all mammals, the extent of development at birth depends on the period of intrauterine development as the factors responsible for fetal development are provided by the maternal placenta and amniotic fluid (Ferner et al., 2009, Fowden, 1995, Fowden and Forhead, 2009, Sibley et al., 1997). Subsequently the eutherian neonate has mature organs for normal function. In contrast, development of most organs at birth in marsupials is significantly less advanced (Setiati, 1986, Szdzuy et al., 2008, Wilkes and Janssens, 1986). From an evolutionary perspective, the survival of these primitive mammalian progeny would have been at greater risk of extinction, but these mammals have adapted a unique lactation system to support postnatal development of their progeny (Daniel, 1993, Goldman, 2002). This development appears to rely on the factors provided through milk (Nicholas et al., 1997). Therefore the marsupial provides an alternative and unique model to understand the process of lung development and also to investigate the potential significance of milk bioactives for signalling this development. Recently we attempted to address the hypothesis of milk regulating postnatal lung maturation by culturing mouse embryonic lungs with tammar milk whey collected early in lactation (Modepalli et al., 2015). The results showed branching morphogenesis and growth of lung explants cultured with milk from day 20 lactation whey was restricted. In contrast, when an embryonic lung was cultured with milk collected between day 40 and 100 extensive branching morphogenesis and tissue growth was observed, and this effect was significantly reduced with milk collected after day 100 of lactation (Modepalli et al., 2015). This suggested that tammar milk collected prior to day 20–25 lacked the factors necessary for programming growth and development of lung, and indeed may have included factors that halt lung progression, indicating that the marsupial milk may contain both positive and negative factors to regulate lung maturation.

In the present study we have exploited the tammar wallaby as a model to better understand the potential role of milk in postnatal lung development of pouch young. To address this aim we have adopted a cross-fostering technique of transferring the pouch young at day 25 of age to a series of mothers at day 15 of lactation so that the young only receive milk from day 15 to 25 of lactation for a period of 20 days. The lungs were then analysed to determine if development was reduced. This regime therefore examined the hypothesis of temporal effects of milk composition on lung development.

## 2 Results

### 2.1 Cross-fostering the PYs to control the milk composition

**Fig. 1:**
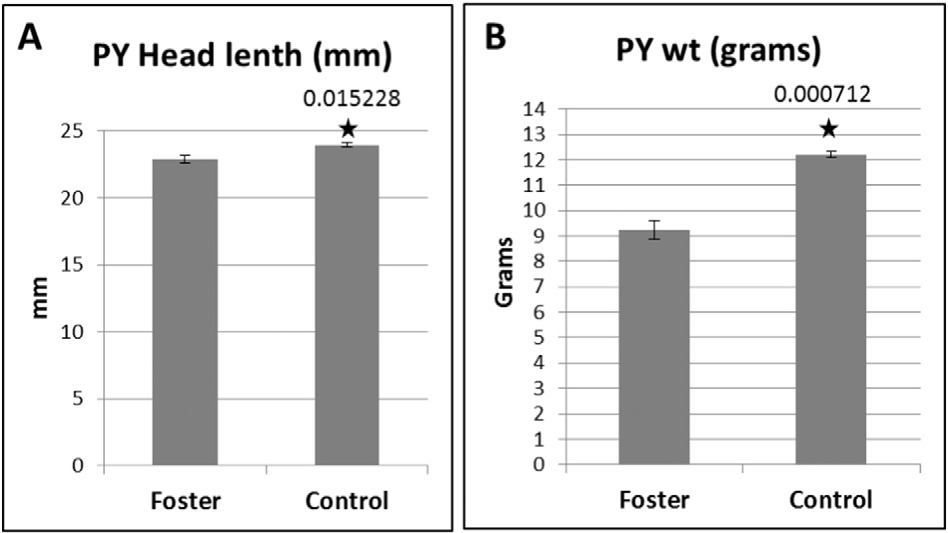
The (A) head length and (B) body weight of foster and control pouch young. Data shown are mean ± SEM for 3 PY from each group. Statistically significant *P* values (< 0.05) are shown with an asterisk.

The day 45 old PY were collected from both foster and control group mothers and individual PY weight and head length were measured and compared between both groups. The fostered PY were significantly reduced in head length (*P* value 0.051228) (Fig. 1A) and weight (*P* value 0.000712) (Fig. 1B) compare to the control PY.

### 2.2 Lung morphology of PY in foster and control groups

**Fig. 2:**
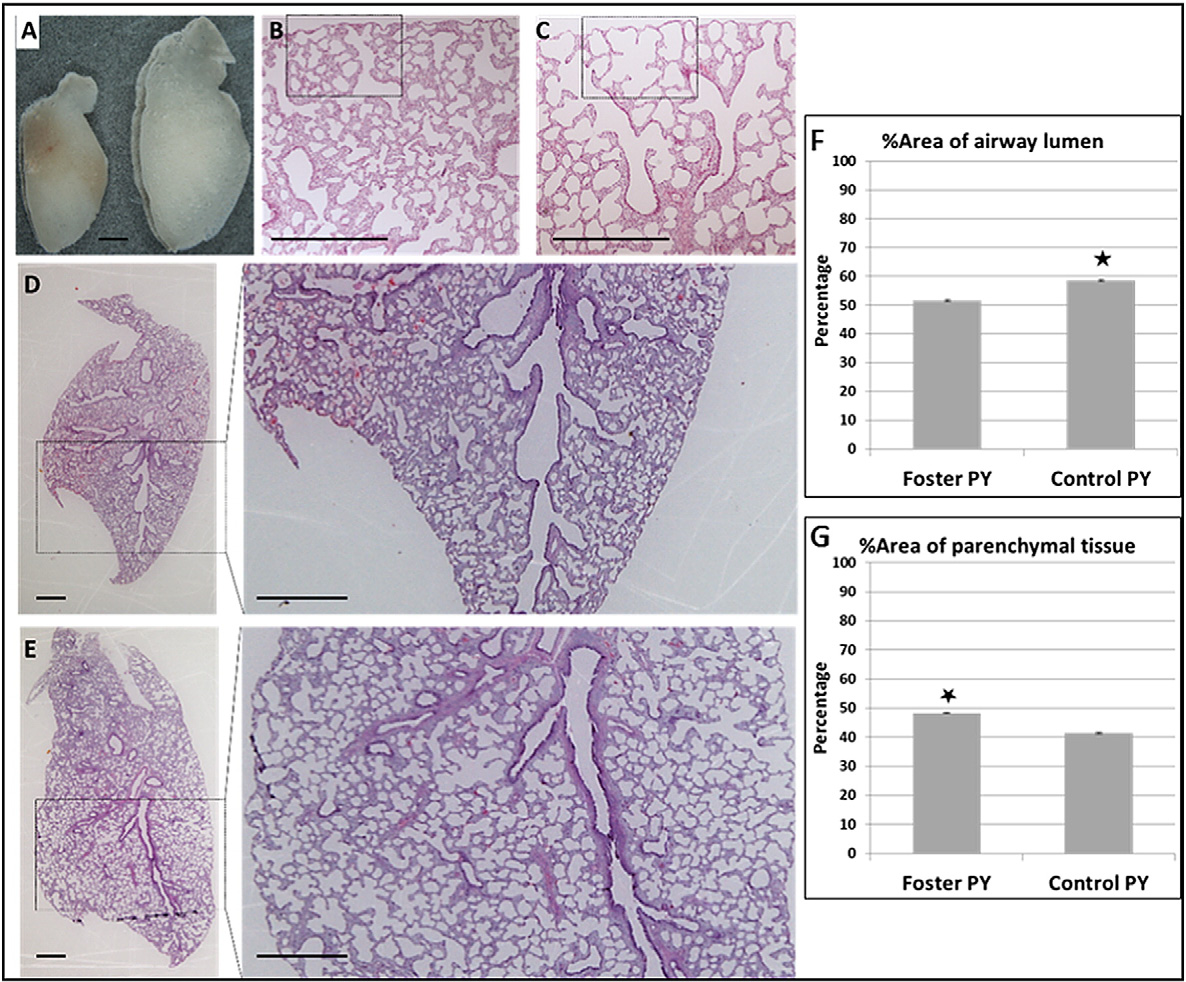
Lung morphology in foster and control PY. (A) The lobe of the left lung from foster (left) and control PY (right). (B–E) Lung sections from fostered and control tammar PY at age 45. Sections were stained with H&E. Inserts show respiratory area in higher magnification, (B & D) the lung parenchyma of fostered PY is denser than those of (C & E) control PY lungs. (F & G) Lung morphological analysis, the percentage of lung composed of parenchyma tissue and respiratory area (alveolar sacs) were measured using Image J. The percentage of area of parenchyma tissue is higher in fostered PY lung and the percentage of area of alveolar sacs is higher in lungs from control PY. Data shown are either representative or the combined mean ± SEM of 3 PY from each group. *P* < 0.05. Scale bar 1 mm.

The size of the left lobe of the lung was assessed and morphological analyses was performed on the right lobe collected from each PY. The lungs were sectioned and stained to assess morphological development and the images were analysed using Image J software. The percentage of parenchymal tissue in lung from fostered PY was significantly higher and the percentage of respiratory lumen area was significantly lower (Fig. 2).

### 2.3 Developmental marker gene expression in lungs from control and foster pouch young

**Fig. 3:**
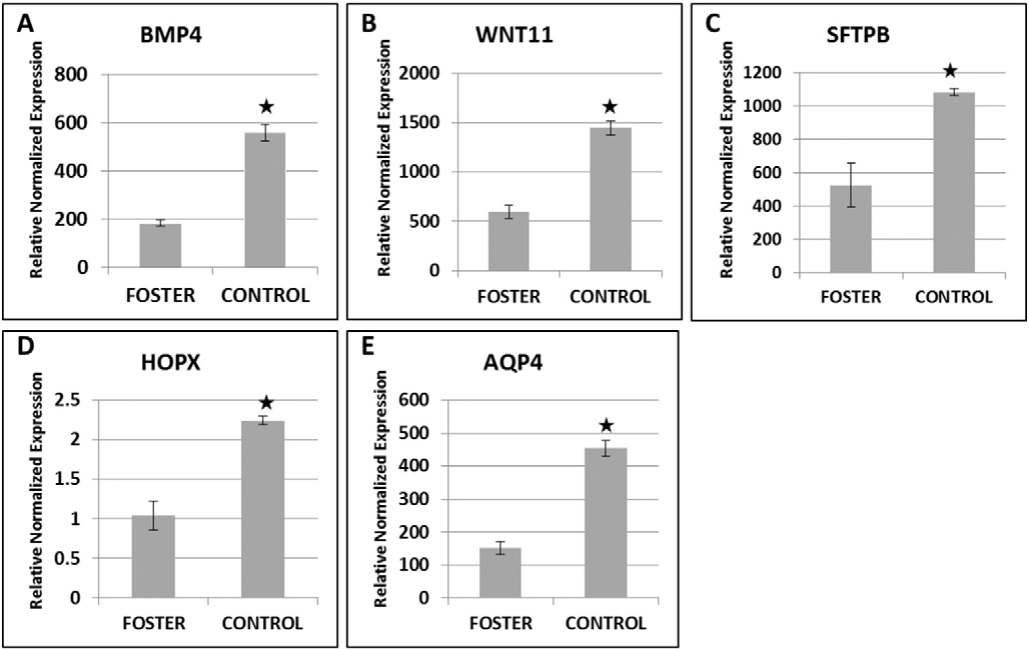
Expression of marker genes for lung development in PY from foster and control groups. RNA was isolated from lungs and the expression of marker genes measured by qPCR. (A) BMP4 & (B) WNT11 (branching morphogenesis), (C) SFTPB (Type-II epithelial cells), (D) HOPX (Type-I epithelial cells) and (E) AQP4 (terminal and airway epithelia). The level of BMP-4, WNT11, SFTPB, HOPX and AQP4 mRNA was significantly increased in control animals compared to foster animals. Data shown are mean ± SEM of 3 PY from each group. Statistically significant *P* values (*P* < 0.05) are shown with an asterisk.

Comparative analysis of expression of genes for developmental markers related to postnatal lung maturation (alveolization and branching morphogenesis) was assessed by RT-PCR analysis (Fig. 3). Results showed that marker genes related to branching morphogenesis (BMP4 and WNT11), alveolization or Type-I epithelial (HOPX), Type-II epithelial (SPB), terminal and airway epithelia (AQP4) showed a significant reduction in lungs from fostered PY compared to lungs from control PY.

### 2.4 Milk composition - total carbohydrates, lipids and protein

**Fig. 4:**
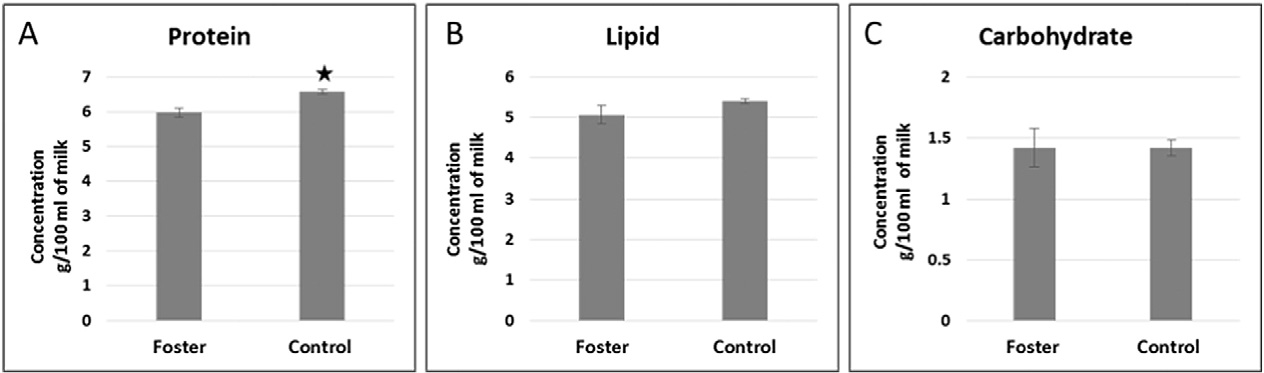
Milk collected from foster and control mothers after final removal of PY to analyse total (A) protein, (B) lipid and (C) carbohydrates. The concentration of protein was significantly higher in milk collected from control mothers compared to the foster group. Data shown are mean ± SEM of 3 PY from each group. Statistically significant *P* values (< 0.05) are shown with an asterisk.

The milk samples collected at the time the PY were sacrificed from both groups were analysed for concentration of total carbohydrates, lipids and protein (Fig. 4). There was no difference in the concentration of total carbohydrate and lipid in milk provided to the PY from both foster and control PY. In contrast, the total protein concentration was significantly lower in the fostered group milk samples.

### 2.5 Identifying secreted milk proteins during early lactation and analysed through functional categorisation

**Fig. 5:**
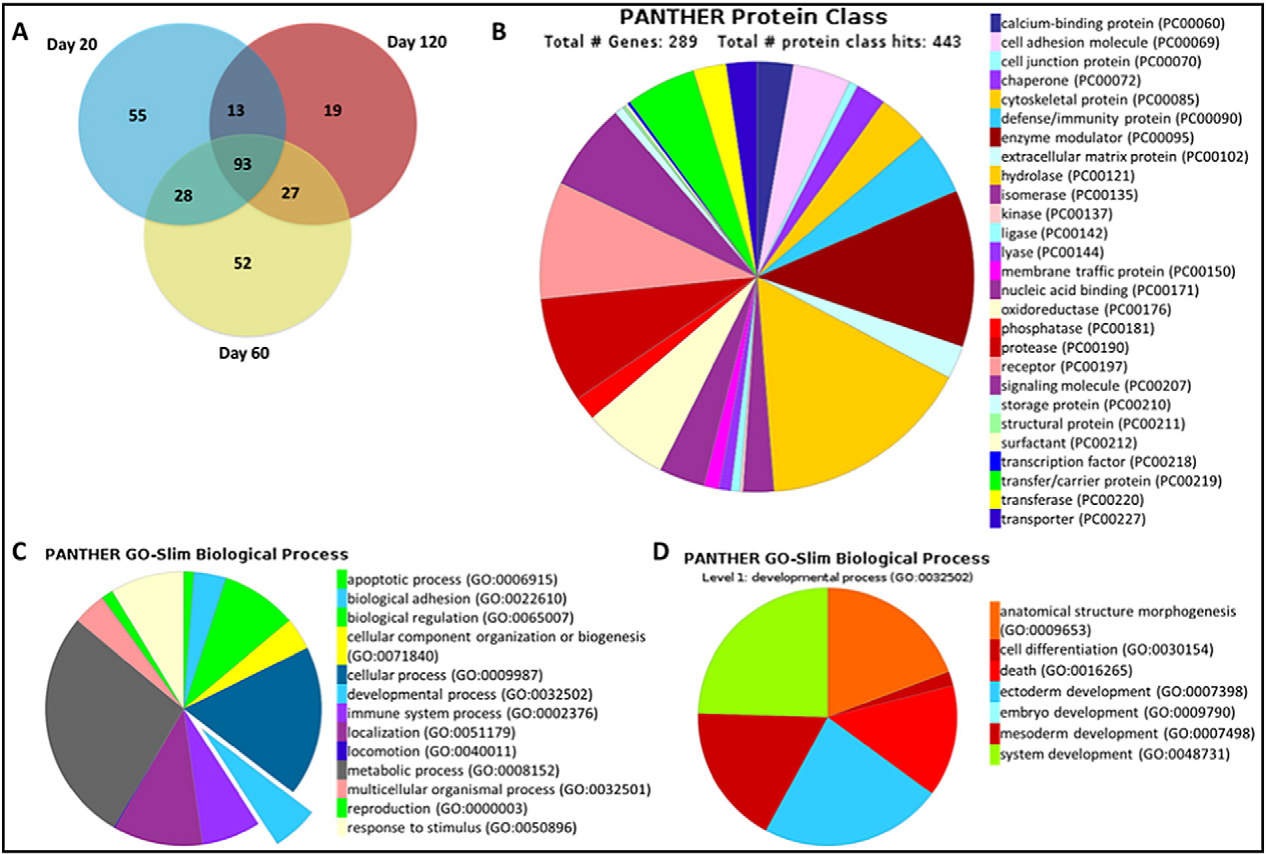
Functional categorisation of tammar milk proteins identified through LC-MS/MS. (A) Venn diagram showing the number of overlapping proteins among milk samples collected from day 20, day 60 and day 120 lactation. (B) Functional annotation of milk proteins from all three time points based on protein class. (C) Functional annotation of secreted milk proteins based on biological process. (D) Sub-classification protein identified under developmental process.

Mass spectrometry was used to identify the secreted milk proteins in milk at day 20, day 60 and day 120 lactation. Using data analysis from LC-MS/MS we identify 189, 200 and 152 proteins in milk collected from day 20, day 60 and day 120 respectively. Among these identified proteins, 93 were reported all three time points. In day 20 milk samples 55 proteins from a total of 189 were uniquely expressed only in day 20 milk sample and 28 were expressed only in milk at day 20 and day 60. Analysis of the 200 proteins reported in day 60 milk samples, 52 proteins were uniquely expressed in day 60 milk, and 27 proteins were only expressed in milk from day 60 and day 120 of lactation. In day 120 milk samples we identified 19 proteins expressed only in this sample and 13 proteins were present in both day 20 and day 120 milk samples. The data are summarised in a pie chart (Fig. 5A) and the list of these overlapping and uniquely reported proteins were presented in the Supplementary data (Table S1).

In order to identify the potential candidates involved in lung development we performed functional categorisation on proteins identified from all three time points (day 20, day 60 and day 120) by using PANTHER™ Classification System. A total of 289 proteins were significantly identified from LC-MS/MS analysis, the details of these proteins were presented in the Supplementary table (Table S1). The proteins were primarily analysed for different functional categories including, biological process, molecular function, protein class, cellular component and pathways, the list of proteins with their related functional categories were presented in Supplementary data Supplementary table (Table S2). The analysis was focused on proteins listed in signalling development (Fig. 5C, D), and the majority of these proteins were involved in system development related to various organs. A number of these proteins were also related to embryonic development (ectoderm and mesoderm) and cell differentiation (Fig. 5D). We also noted that many of these proteins were involved in regulating cell behavior in terms of differentiation, morphogenesis, cell-cell signalling, growth and transition of epithelial to mesenchymal cells, specialized features necessary in development process of the respiratory system. Some proteins were identified as involved in embryonic organ development of eutherians including signalling lung maturation.

## 3 Discussion

### 3.1 Tammar lactation is potentially programmed to regulate the developmental changes in pouch young

The tammar is born after a short gestation (~ 28 days) and the lungs were immature and still at the canalicular stage of development, similar to the eutherian fetal lung (Runciman et al., 1998). The majority of development occurs during early postnatal life (Frappell and MacFarlane, 2006, Saunders et al., 1989, Wang et al., 2009). In contrast, in the majority of eutherian species, lung develops prior to birth, particularly in response to the placenta which regulates intrauterine fetal development by supplying various bioactives (Bell et al., 1999, Garnica and Chan, 1996) supporting fetal respiration (Sibley et al., 1997). Unlike eutherians, the marsupial newborn development relies on factors supplied through milk (Nicholas et al., 1997, Sharp et al., 2014). Based on our previous study (Modepalli et al., 2015), we observed the milk protein collected from different time points of early lactation had temporally related effects on embryonic mouse lung *in vitro*. Based on this observation we speculated that tammar milk secreted before ~ day 20–25 lacks the necessary factors for stimulating lung development whereas the milk secreted between 25 and 100 days post-partum stimulates normal lung development *in vitro* (Modepalli et al., 2015). In the present study we have adopted a cross-fostering technique to assess effects of milk on postnatal lung development of the PY.

The histological analysis of lung from fostered PY exposed only to milk with a composition seen prior to day 25 of lactation showed immature morphology compared with lung from control animals, in terms of the total area of respiratory lumen and the presence of more intermediate tissue with thicker septa. This morphology indicated that the immature lungs had less area for gaseous exchange and also the thicker septal structures would have reduced gaseous exchange (Fraser et al., 2004, Weibel, 1999, Weibel, 2009). In addition, these data were consistent with the lower expression of marker genes in fostered PY lung indicating a likely delay in lung development. For example, the expression of both BMP4 and WNT11 genes was lower and these genes were known to be involved in various aspects of branching morphogenesis of lung and other organs like kidney (Bellusci et al., 1996, Pongracz and Stockley, 2006, Wodarz and Nusse, 1998). In addition, the reduction in expression of AQP4, a marker gene predominantly expressed in the respiratory airway epithelia cell of peripheral lung region (Song et al., 2000), was also consistent with a delay in branching morphogenesis. Similarly, significantly reduced expression of the marker genes related to alveolization (HOPX and SPB) in fostered PY lungs indicated both Type-I and Type-II epithelial cells that produce surfactant proteins SPB were reduced (Treutlein et al., 2014). These data were also consistent with reduced expression of the transcriptional factor HOPX involved in lung maturation through alveolization (Yin et al., 2006) which is also specifically expressed in the Type-I epithelial cells (Treutlein et al., 2014). Taken collectively, the data indicated poor alveolization and lung maturation in fostered PY.

Previous experiments have shown day 20 milk either lacks the necessary signalling factors for stimulating embryonic lung development or alternatively may include factors to reduce lung development until the PY reaches more than 20 days of age (Modepalli et al., 2015). On the other hand, the control group PY had normal lung growth, which suggests the factors responsible for lung maturation were present in milk secreted beyond day 25 of lactation. This data also correlates with morphological changes in lung observed in pouch young over this period, where the lung displays a slow rate of septal formation (Modepalli et al., 2015, Szdzuy et al., 2008). Based on the lung morphological analysis during the early postnatal life of tammar pouch young, an increase of alveolization in lung was observed from 30 days of age and which continued until 100 days (Modepalli et al., 2015, Runciman et al., 1998, Runciman et al., 1999, Szdzuy et al., 2008). In addition, from our previous studies we observed that an embryonic lung cultured in day 40 milk showed extensive branching morphogenesis and tissue growth. These observations were consistent with the hypothesis that the marsupial tammar milk drives the developmental process in pouch young (Nicholas et al., 1997, Sharp et al., 2009, Sharp et al., 2016).

### 3.2 Maternal signalling factors secreted by mammary gland into milk may influence lung maturation of pouch young

During fetal development the lung undergoes extensive morphological changes and these processes are regulated by both intrinsic and extrinsic factors, including maternal growth factors and hormones (Ferner et al., 2009, Fowden, 1995, Fowden and Forhead, 2009, Sibley et al., 1997). One of the key focuses of this study was to use mass spectrometry to identify candidates as potential signalling factors secreted in milk during tammar lactation. In order to identify the putative protein involved in lung development we perform mass-spec analysis on milk whey fraction collected during day 20, 60 and 120 lactation. A number of growth factors were reported to influence the fetal lung development, such as platelet-derived growth factor alpha polypeptide (PDGFA), insulin-like growth factor 1 (IGF1) and connective tissue growth factor (CTGF) (Baker et al., 1993, Friedrichsen et al., 2003, Hoch and Soriano, 2003). From the present analysis, potential bioactives secreted in tammar milk include PDGF, IGFBP5, IGFBPL1 and EGFL6. Based on the literature we speculate that some of these factors may be involved in signalling specific lung developmental changes in pouch young. The PDGFs are known to influence lung development by regulating alveolization during alveolar stage of development; gene knockdown studies have demonstrated that PDGFA helps in recruiting smooth muscle cells to form alveoli and septation (Boström et al., 2002, Boström et al., 1996). From proteomic analysis we identified PDGFA in milk at all three time points that correlated with increasing septation and alveolization in lung, during early postnatal life in the pouch young (Modepalli et al., 2015). The role of insulin-like growth factor (IGF) binding protein in lung development is well studied (Spencer et al., 1995) and from the present data we observed IGFBP5 and IGFBPL1 were secreted in milk. IGFBP are temporally expressed during different stages of lung development and specifically during saccular and alveolar stages of lung development (Wallen et al., 1997). IGFBP5 induced the epithelial-mesenchymal transition and fibroblast activation to increase extracellular matrix deposition (Pilewski et al., 2005, Yasuoka et al., 2006). IGFBPL1 is known to interact with IGFs and modulate the growth effect of IGFs (Cai et al., 2005). Although the function of IGFBPL1 have not been clarified its role in lung development, with respect to present study IGFBPL1 may interact with IGF and regulate its function. Members of the epidermal growth factor (EGF) repeat superfamily genes are often involve in regulating cell proliferation (Yeung et al., 1999). EGFL6 is expressed in all major fetal tissues and generally, absent in normal adults tissues, except in tumour of lung and brain (Yeung et al., 1999). However, the role of EGFL6 in fetal lung development is unknown, although recent studies reported that EGFL6 was involved in angiogenesis during bone development (Chim et al., 2011) which may be consistent with a role in fetal lung development related to angiogenesis.

The interruption in regular development that affected pouch young development in fostered pouch young is similar to a human preterm neonate born with respiratory disorders due to interruption of the regular development processes that results potentially from reduced exposure to maternal signalling factors (Fraser et al., 2004, Swischuk, 1997). Similarly, as the marsupial is born with immature lungs and the majority of development is entirely dependent on postnatal milk consumption, the disruption of the correct milk factors effects lung maturation. Based on the present study, an interruption in the regular consumption of milk secreted from the appropriate stage of the lactation period has resulted in delayed lung development. Hence, this demonstrates how, in marsupial lactation, the continuous change in milk composition (Nicholas et al., 1997, Sharp et al., 2009) regulates developmental changes in the pouch young. The marsupial model therefore provides new opportunities to identify factors supplied through milk that impact on lung development. Further, the milk proteomic analysis presented here demonstrates that tammar milk may have significant influence on regulating the lung maturation of pouch young during lactation by providing those necessary signalling factors through milk.

## 4 Experimental procedures

### 4.1 Ethical approval

The colony of tammar wallabies (*M. eugenii*) used for experiments was maintained at CSIRO Ecosystem Sciences, Canberra, Australia. All animal experimentation was approved by The Deakin University and CSIRO Animal Ethics committees.

### 4.2 Cross-fostering

**Fig. 6:**
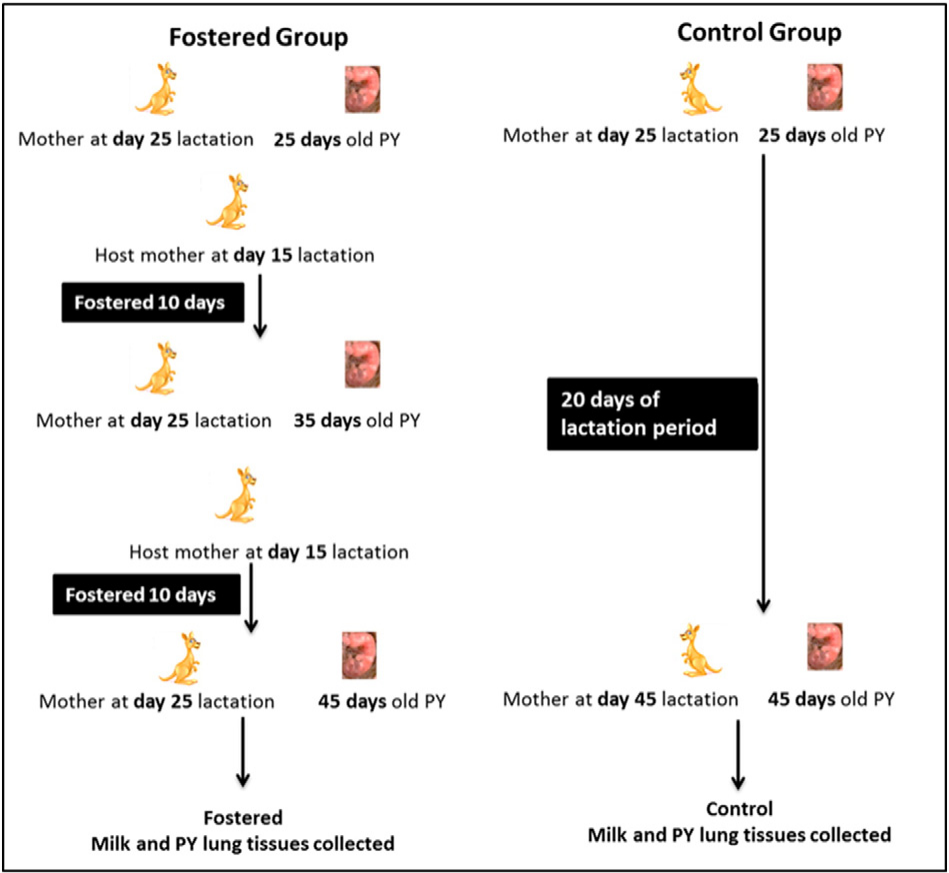
Schematic representation of the cross-fostering experimental design. In the fostered group, to restrict the PYs < 25 days lactation milk composition, the PY at age 25 days was transferred to a day 15 lactating host mother. After 10 days the PY was 35 days of age and the mother progressed to 25 days of lactation. The 35 day old PY was again transferred to a day 15 lactating mother. After 10 days the PY was 45 days of age and the mother was at 25 days of lactation. The control group PYs remained with their original mother until 45 days of postnatal life.

The age of pouch young was either determined from known birth dates or estimated by measuring head length (Poole et al., 1991). The pouch young were removed from the teat of their original mother and transferred to the respective host mother by gently directing the teat into the mouth of the pouch young. The host mother was released into the animal yard and placed under regular observation. The control pouch young at approximately day 45 of age were removed from the mother and the age confirmed by measuring the head length. For cross-fostering, initially the pouch young were allowed to grow to day 25 after birth with the original mother. The PY (pouch young) was detached from the mother and transferred to a host mother at day 15 lactation. After allowing for 10 days of fostering the pouch young was once again removed for fostering to a day 15 lactating host mother. This maintained the pouch young with milk composition not extending beyond day 25 for 45 days of postnatal life (Fig. 6). Each pouch young was evaluated by measuring the head length and body weight. The lung was dissected from the young, the right lob was separated and frozen immediately at − 80 °C for RNA analysis and the remaining part of lung was rinsed thoroughly with sterile PBS and fixed overnight in 4% formaldehyde prior to paraffin embedding. Tissue sections (5–6 μm) were prepared and stained with haematoxylin and eosin (H&E) for examination of morphological development. Other internal organs were also collected for morphological analysis.

### 4.3 Morphological analysis of embryonic lung

**Supplementary Fig. S1:**
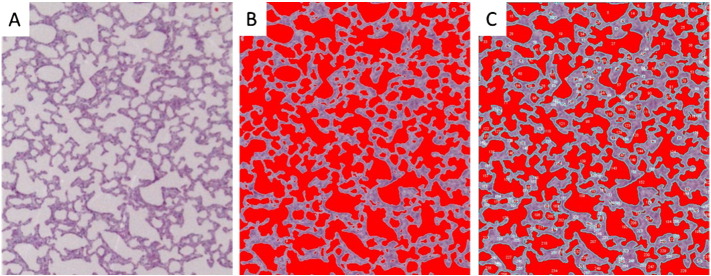
Lung tissue section morphological analysis using Image-J software. (A) A lung tissue section H&E stained. (B) The image was adjusted using the colour threshold. (C) Overlaying the outlines of the sacs using the “Analysed Particles” method.

Histological analysis was performed by serial sectioning of fixed lung and staining with haematoxylin/eosin (Fig. S1A). These analyses were performed on an average of 100 images from each experimental group using Image-J software. Each image was adjusted using the colour threshold default method (Fig. S1B). The sacs were analysed by overlaying the outlines of the sacs using the “Analysed Particles” method (Fig. S1C). The default setting included, size (pixel^2) ranging from 0 to infinity and circularity ranging from 0.00 to 1.00. The summary of results of each image were collected and included count (number of alveolar saccules per slice), average size (average size of airway lumen) and % area (percentage of area covered by airway lumen and parenchymal tissue). The data collected from each image was transferred to Excel for statistical analysis. Results were analysed using a 2 tailed, Type-2 *t*-test to estimate the statistical significance and the error bars indicates mean ± SEM.

### 4.4 Total RNA isolation and quantitative RT-PCR

Total RNA was extracted from lung tissue using a PureLink® RNA Mini Kit (Life Technologies) following the manufacturer's instructions and subsequently quantified by spectrophotometry (Nano drop ND-1000, Biolab, VIC, Australia). First-strand cDNA was synthesised using Superscript III™ Reverse Transcriptase (Invitrogen), following the manufacturer's instructions. Quantitative RT-PCR (qPCR) was performed using SsoFast EvaGreen Supermix (Bio-Rad) and CFX96TM Real-Time PCR Detection System (Bio-Rad). The PCR reaction (20 μL) contained 1 × master mix, 0.25 μm of forward and reverse primers (Table S3) and diluted cDNA template. All samples were assayed in triplicate. Amplification curves were generated with an initial denaturing step for 30 min at 94 °C, followed by 40 cycles of 94 °C for 30 s, 60 °C for 30 s and 72 °C for 30 s. The GUSB and 18S genes were used as an internal control.

### 4.5 Milk collection and analysis

Milk samples were collected from both control and fostered groups after removing the PY. Tammars were anaesthetised with 1% isoflurane and 0.2 IU of Oxytocin-S® (Intervet, Boxmeer, The Netherlands) administered intramuscularly prior to milk collection. Approximately 100–200 μL of milk was collected from each animal by applying gentle pressure to the mammary glands and milk was stored at − 80 °C until further analysis. Milk samples were analysed for total protein, carbohydrates and lipid content. The total protein content was measured using Micro BCA Protein Assay Kit (Thermo Scientific™ Pierce™ Micro BCA™ Protein Assay). The carbohydrates was analysed by measuring the total hexose against standard glucose (Messer et al., 1988) and the lipid by measuring the total essential fatty acids against standard oleic acid (Atwood and Hartmann, 1992). All measurements fell within the range of the standard curves.

### 4.6 Mass-spectrometry

In order to identify the secreted protein in milk from day 20, 70 and 130 lactation, the total protein of 200 μg of each sample were run on 12.5% polyacrylamide gel to separate the protein into 26 fractions based on the mass. Bands were washed with 50% acetonitrile in 25 mM NH_4_HCO_3_ and subjected to trypsin digestion overnight at 37 °C, the mixture was analysed by mass spectrometry. The raw mass spectrometry data was analysed using MASCOT software (Matrix Science, London, UK) to search for peptide fingerprints. The protein search was performed using *Macropus* *eugenii* transcriptome data, protein score greater than 75 are considered to be significant (*P* < 0.05) and proteins with minimum of 2 peptide match was considered.

### 4.7 Functional categorisation

The proteins were categorised based on their biological function and molecular process using PANTHER™ Classification System (http://www.pantherdb.org/). The listed proteins were functionally classified and presented in pie chart with number of proteins involved in each function.

The following are the supplementary data related to this article.

Supplementary Table S1 Table detailing the list of proteins significantly identified from LC-MS/MS analysis and the list of proteins reported in pie chart. Supplementary Table S1

Supplementary Table S2 Table detailing the list of proteins analysed through functional categorisation. Supplementary Table S2

Supplementary Table S3 Primer sequences used for mRNA quantification by RT-PCR. Supplementary Table S3
